# Global Biobank Meta-analysis Initiative: How can global health benefit by its use?

**DOI:** 10.7189/jogh.13.03054

**Published:** 2023-10-27

**Authors:** Elena V Alpeeva, Konstantin S Sharov

**Affiliations:** Koltzov Institute of Developmental Biology of Russian Academy of Sciences, Moscow, Russia

## HUMAN GENETIC CRYOBIOBANKS AS AN INFORMATION RESOURCE FOR MEDICINE

Cryogenic banks of human biological material (blood and other biological liquids, tissues, organs, gametes, etc.) may be a convenient tool for global health initiatives. Besides their direct use in blood transfusion, organ transplantation, burn therapy, clinical surgery, in-vitro fertilisation and other medical applications, they allow us to collect, systematise and analyse large sets of human genetic data [1,2]. These data may be later used for receiving new insights into understanding the nature and origin of human diseases, and their treatment. They also allow a novel perspective on inheritance of pathologies.

Collections of human cells can also be considered as the genetic biobanks as they are used not only as a cell storage but as a means of genetic information preservation and a valuable tool for the investigation of genetic diversity and epigenetic data. The genetic information on the cells in the collection is usually analysed using karyotype description techniques and short tandem repeat (STR)-profiling to create the cell culture data sheet for each cell culture which is stored. Different cells within the collection can easily be subjected to the gene editing manipulations and provided to the users to enable a broad range of studies. One of the new possibilities to reverse the fate of differentiated “adult” cells is the method of obtaining induced pluripotent stem cells (IPSCs) with the epigenetic status close to the stem cells of the embryo. Induced pluripotent stem cells can be then directed to different routes of differentiation and thus used to investigate the epigenetic mechanisms and various forces guiding the development of tissues and organs of a human organism normally or in the case of malformation or pathology.

All this is usually done via so-called genome-wide association studies (GWAS) of human biological material stored in biobanks. Full sequencing and creating different “omic” (genomic, proteomic, transcriptomic, metabolomics, metagenomics, and phenomics) databases are usually done in large biobanks [3]. They perform large-scale studies of genomic associations that make it possible to correlate certain genes with the phenotypic traits (in our case pathologies) of a person, group of relatives, ethnic group or an isolated community. Among numerous applications in global health, the GWAS methodology may provide valuable results in epidemiology, oncological studies, pharmacology, senescence investigations, apoptosis, and inherited diseases studies. Besides, GWAS of human cryobiobanks can give an even more impressive outcome, if the medical studies are combined with anthropological research [4]. For example, if an in embryo genetic study has been performed, GWAS enable one to assess the probabilities of developing various diseases in a future person. Another example is tracing ancient routes of migrations and interethnic/interracial genetic relations. This information can potentially shed light on how we could treat diseases most effectively in different social groups [5].

## EMERGENCE OF GLOBAL BIOBANK META-ANALYSIS INITIATIVE

Global Biobank Meta-Analysis Initiative (GBMI, https://www.globalbiobankmeta.org) has recently (2019) emerged as an international GWAS project. As of April 2023, GBMI combined digital data from 24 national cryobanks of biological material representing 15 countries (in alphabetical order): Australia, Canada, China, Estonia, Finland, Iceland, Japan, Republic of Korea, the Netherlands, Norway, Qatar, Taiwan, Uganda, UK, and USA [6]. As the GBMI’s authors claim, around 2.2 million genotype samples have already been researched with approximately 70 million genetic variants in total and 40 million variants tested in two or more biobanks [6,7].

The purpose of this opinion piece is to analyse GBMI in the context of usefulness and applicability for health specialists. In what does GBMI differ from peer GWAS projects that have been already used in global health?

1. Truly huge size;

2. Potential for genetic statistic-based fine mapping;

3. Statistic-based post-GWAS quality control;

4. High level of international collaboration;

5. Wide spectrum of diseases is being studied, that are traditionally regarded inherited (e.g. asthma) and not inherited (e.g. heart failure, cerebrovascular accident or appendicitis);

6. Genetic investigation of insufficiently studied diseases is possible;

7. Metadata, including social and anthropological information, is available for a considerable number of samples;

8. Considerable ancestry diversity: around 27% of GBMI samples are of non-European origin;

9. Low- and middle-income countries are included in the research;

10. Phenotypes are being curated and harmonised;

11. Opportunity for additional validation across various cryobiobanks for a large part of samples;

12. Multiple sex-specific associations have been established for different diseases that allow a new look at them;

13. Proteome-wide Mendelian randomisation in several proteome-wide association studies (PWAS) have been performed;

14. Potential for multi-ancestry and meta-analytic transcriptome-wide association studies (TWAS).

## DISEASES UNDER INVESTIGATION

The primary goal of disease studies within the GBMI project was to evaluate the inheritance factor in the diseases. The secondary tasks were to systematise data for genetic anthropological investigations, assess polygenic risks of diseases development, study comorbidity and pleiotropy and consider prospects of genetic therapy of diseases. **Table 1** summarises the features most interesting for health specialists. Besides, **Table 1** offers ways of improvement of GBMI to use in health projects more efficiently.

**Table 1:** Potential use of GBMI by health workers, including researchers and practitioners

Feature	What is already done and applicable for use in global health	What may be added/improved/corrected for use in global health
Diseases studied within the GBMI	The following diseases are currently under investigation in GBMI: 1. asthma; 2. COPD; 3. heart failure; 4. stroke (cerebrovascular accident); 5. gout; 6. venous thromboembolism; 7. POAG; 8. abdominal aortic aneurysm; 9. idiopathic pulmonary fibrosis; 10. thyroid cancer; 11. hypertrophic cardiomyopathy; 12. uterine cancer; 13. acute appendicitis.	The following diseases are ready to be researched in GBMI: 1. diabetes; 2. schizophrenia; 3. rheumatoid arthritis; 4. obesity; 5. myopathy; 6. colour blindness; 7. monochromacy; 8. haemophilia. The list of diseases may be significantly increased. Different groups of diseases should be clearly discriminated between regarding the importance of inheritance factor.
Wide-association studies	Genome sequencing is full in many biobanks within the GBMI	TWAS and PWAS need be made and cross-validated across several biobanks
Assessing genetic therapy of diseases	Drug discovery is given top priority	Too strong emphasis on *in embryo* treatment should be avoided
Validating genetic findings	Proprietary SLALOM, a summary statistics-based quality-control method, is developed for fine mapping [7]. Suspicious loci are identified	Additional cross-validation across the greater number of biobanks, both those which are included in GBMI and the outside ones, is needed
Gene properties	For some phenotypes, polygenic risks, comorbidity and pleiotropy are researched	Penetrance and gene expressivity should be studied and made allowance for. Little frequency of occurrence (or low gene expressivity) along with insufficient amount of samples should be paid a special attention to avoid unreliable results
Phenotype curation	It is made according to the ICD codes to PheCodes for diseases and using OPCS codes for procedures [8]	Filling gaps in phenotype definitions by ICD- or OPCS-based restoration may lead to inconsistent and even wrong results in some cases, especially for rare diseases with little amount of corresponding samples, or complex cases with large degree of pleiotropy and/or large number of comorbidities. More thorough approaches to defining phenotypes should be included in the set of techniques that should be based upon more detailed metadata
Medicine-related ancestry studies	Some anthropological research is being done. Around ¼ of the full genome set is comprised by the samples of non-European origin: Latin American (0.9%); African (2.2%); Middle and South Asian (1.6%); Far East (21.6%) and Near East (0.7%) [6].	A deeper enquiry into origin-specific (e.g. ethnicity-specific, race-specific, or community-specific) diseases is necessary. Genetic data of isolated communities representatives must be included in the set. This is crucial for understanding nature of diseases of isolated communities and diseases of progeny of cross-relation marriages. Migration routes should be researched to understand ways of historical spreading the diseases. More samples of non-European and non-White origin should be added to study origin-specific pathologies. Greater geographic representation should be maintained, as now the majority of samples is constituted merely by the US geographical and national origin.
Metadata	Some samples have supplementing metadata about persons.	Large gaps in metadata for other samples should be gradually filled. Otherwise these samples should be used for disease phenotype description with care. New samples included in cryobiobanks should have a comprehensive list of supplementing metadata. Behavioural metadata are necessary for some diseases studies (e.g. psychiatric and neurological disorders) [9] that are now totally missing in GBMI.
Availability of data	Accessible for participants of the programme	It may be wise to make GBMI data fully accessible for researchers and medical workers outside the programme, e.g. as a free-of-charge genetic, proteome, transcriptome, biochemistry, molecular biology and metadata cloud service or database. For health practitioners, a special database or software may be developed. It may not contain detailed genetic data, but it must contain health-specific information on phenotypes, as well as recommendations for diagnosing and treatment

GBMI – Global Biobank Meta-Analysis Initiative, COPD – chronic obstructive pulmonary disease, POAG – primary open-angle glaucoma, SLALOM – StatisticaL Analysis of Locus Overlap Method, TWAS – transcriptome-wide association studies, PWAS – proteome-wide association studies, ICD – International Classification of Diseases, PheCodes – Codes of Phenotypes Database, OPCS – Office of Population Censuses and Surveys Classification of Interventions and Procedures

## BENEFITS FOR GLOBAL HEALTH

To be sure, GBMI does provide enormous opportunities for global health, both scientific investigation and practice. Several factors contributed to this.

First, there is no need in complex procedures to collect field data, as GBMI cryobiobanks contain human genetic material for research in an easily accessible form.

Second, the costs of sequencing and performing PWAS/TWAS are relatively low in GBMI due to the huge size of the project and involvement of internationally funded institutions.

Third, there are familial cases where samples are taken from persons of several generations (maximum three generations of relatives as for now). That allows one to study the inheritance factor of diseases in close relations.

Fourth, data from multiple biobanks are combined and accessible via the same network.

Fifth, GBMI project contains data in a standardised form and therefore it is readily scalable. New biobanks may be added to the project after the applications of the corresponding research groups have been approved and necessary IT/technical steps have been done. GBMI may be regarded as a potential forerunner of a worldwide global biobank, should such a biobank be created in the future. Health researchers will be able to add their institutional biobanks to GBMI and test the genetic data of their studies against the GBMI database.

## POINTS OF CONCERN

That notwithstanding, there are several important drawbacks (some of them seem to be temporary) in GBMI that should be always taken into account by health scientists and practitioners which would opt to use GBMI functionality. They are briefly discussed below.

1. Lack of dependable metadata. For the majority of samples, there are no comprehensive clinical metadata. For those samples that do contain health-related metadata, incomplete information is present which is, in most cases, a mere statement of diagnosis without anamnesis or detailed description of clinical treatment/interventions. This adds almost nothing to the results of genetic investigation, as the diseases currently studied within the GBMI project are well-known to geneticists. To research understudied diseases, a new approach to collecting clinical metadata that supplement a sample will be required.

2. Absolutising the inheritance factor in diseases development. GBMI’s community tends to attach too great importance to the factor of inheritance in their diseases research. But there are not many diseases known to humanity that are truly genetic, i.e. inborn.

From the list of currently studied diseases by the GBMI team (**Table 1**), only idiopathic pulmonary fibrosis and hypertrophic cardiomyopathy (the first group) may be called inherited in the true meaning of this term. Expressivity of the corresponding genes is high. This almost always leads to a clear clinical picture of these diseases without significant variations or degrees of manifestation.

Most of diseases that GBMI’s authors include in the list of genetic pathologies, are multi-factor, e.g. asthma, COPD or gout (the second group). Epigenetic factors often play an even more important role in the development of these diseases than genetic ones. Epigenetic factors may give a clue to our understanding the difference in the severity of the course of diseases, i.e. why one person may have a very severe course and another an asymptomatic course [10]. In the prenatal development of a human organism the environment might change the epigenetic regulation of certain genes and, therefore, the phenotype [11,12]. Sterile neonatal conditions void of any pathogens make an adult person immunologically untrained regarding certain causative agents, even the most common ones. This may cause a severe course of the corresponding diseases. Normally, an organism has to be acquainted with different antigens at the neonatal stage. Among the most recent theories of epigenetic influence on a human’s susceptibility to infectious and non-communicable disease are the health concept [13] and prenatal programming hypothesis [14]. Data about prenatal epigenetic factors (e.g. the induced alterations in a foetus development and its ability to adapt to these alterations) can allow us to assess the probability of appearing metabolic and cardiovascular diseases in the postnatal life with greater precision [12]. These diseases, in turn, may be additional risk factors in acquiring an infection and developing high risk of comorbidities, as it is the case for influenzas, parainfluenzas and coronavirus disease 2019 (COVID-19) [15].

Epigenetic factors may lead to the complete absence of a disease in favourable conditions, even though a person has genetic predisposition [10,16]. However, epigenetic factors are not investigated in the GBMI initiative. For their proper investigation, rich metadata about a patient and results of molecular biology studies have to be present. Metadata should include detailed medical history, preferably collected at different age of the person.

The third group of diseases (**Table 1**) may be regarded completely non-inherited, viz., stroke, malignant tumours, or acute appendicitis. GBMI’s authors presumably study genetic predisposition for these diseases, e.g. they assess the size and form of appendix, or project the level of thyroid hormones. This is commendable and is done to broaden the genetic paradigm of our understanding inheritance, but the GBMI researchers do not clearly express their purposes, nor explain their motives. That may be highly misleading for the clinicians that would like to use GBMI data.

The fourth group is comprised by diseases without our clear understanding of their nature (whether inherited or not), e.g. heart failure or glaucoma. GBMI research is, to be sure, helpful here. However – once again – GBMI’s authors do not overtly explain their goal. An outsider researcher may be misled that GBMI allegedly proved the inherited character of these diseases.

A clear discrimination between the four groups of diseases described has to be done to avoid misinterpretations and false conclusions by health specialists. As of now, a strong bias toward inheritance factor is done within the GBMI project.

3. No gene expression data. For complex pathologies, comorbidities, pleiotropic cases, and understudied diseases, clinicians will probably wish to have comprehensive data on penetrance and expressivity of the corresponding alleles. No such information is currently present in the GBMI project. It would be commendable to fill this gap gradually, since penetrance and expressivity information is necessary for modelling diversity of phenotype pools.

4. Another side of curating phenotypes. Despite curating and harmonising phenotypes provide universalism and simplicity convenient for clinicians, they also have negative effects. Many complex disease cases may be neglected in this approach. In short, GBMI’s mass phenotype curation leads to a picture too simplified, too standardised, too smooth. In real life almost any case of inherited disease deserves the detailed description and thorough analysis for planning adequate treatment procedures.

5. Another side of drug discovery. Drug discovery, which is a primary goal of GBMI, is by any means useful for pharmacology and drug therapy. However, *in embryo* treatment that is an important part of genetic treatment, is an ambivalent intervention. On the one hand, without taking into account epigenetic factors, in some cases it is difficult – sometimes almost impossible – to judge about real threat to a future person of a genetic mutation that was detected in his or her embryo. Benefit/risk ratio of the planned genetic intervention is unclear for the baby in such cases. On the other hand, if applied in *in vitro* fertilisation procedures, *in embryo* treatment is sometimes dubious from the bioethical view. Indeed, such a treatment will involve choosing and discarding “bad” or “excess” embryos. GBMI seeks ways of drug discovery and genetic treatment, but does not deliberate on the possible scenarios of clinical application.

**Table 2** compares three large biobanks and highlights their achievements and inferiorities.

**Table 2:** Advantages and inferiorities of three large international biobanks

Parameter	Bioresource centre Riken, Japan [17]	American Type Culture Collection (ATCC), USA [18]	European Collection of Authenticated Cell Cultures (ECACC), United Kingdom [19]
Type of funding	Government-sponsored	NGO	Government-sponsored
Number of biodiversity collections	5	5	2
Laboratory mice	9000	–	–
Plants	840 000	400 species (seeds)	–
Cell cultures	15 600	More than 20 000	+
DNA samples	3 900 000	+	–
Microorganisms	29 000	Around 20 000	+
Backup systems	+	+	+
Concordance to ISO 9001	?	ISO 9001: 2015, ISO 13 485: 2016, ISO 17 025: 2017, SO 17 034: 2016	?
Digital catalogue of specimen	+	+	+
Genetic databases	–	–	–
Genome editing possibilities	+	+	–
Sequencing possibilities	–	+	–
Additional research possibilities	+	+	+
Inclusion into international projects	National Bioresource project	–	?
Inclusion in databases networks	Asian collaborative networks	?	?
Educational options (undergraduate, graduate and post-graduate studies)	+	+	+
Ethical committees	3	Adherence to common bioethical standards	?

NGO – non-governmental organisation, ISO – International Organization for Standardization

## FUTURE POINTS OF DEVELOPMENT OF BIOBANKS

Few current biobanks, even large, contain detailed metadata and time scaling. Life-long collection of patients’ data with rich metadata may be an important point of biobanks development. That information will be definitely of especial importance to medical applications and therapy, as it will demonstrate the progress of diseases course, or, on the contrary, their treatment. In this aspect, biobanks may become the critical sources of relevant data for disease incidence calculation and causality establishment.

Currently, even such projects as GMBI face the problem of protocols discrepancy. That problem prevents a smooth integration of different biobanks in biobank networks and may be a source of potential bias. Besides, not every biobank had a similar focus on a specific trait, thus leading to an uneven study frame. Therefore, data harmonisation and the need for a unified data formats that could be adopted by other studies in an attempt to create a global authoritative resource on genetic data are other important points of biobanks development. Different biobanks’ specialisation may be an advantage, not a drawback. Various biobanks may combine their data and findings collected regarding different race and ethnic groups as well as geographical regions. Other crucial steps of uniting different biobanks in networks are achieving the following opportunities: cross-check in research; simplicity of access; redundancy in storing specimen; and effective data curation.

The artificial intelligence (AI) analysis is a rapidly developing form of biobank-related research [20-22]. In the future, there are reasons to believe that the AI analysis can become a tangible and useful tool not only for performing wide-association studies, but also for detecting the problematic points that prevent uniting different biobanks in biobank networks. It may be also used in pathway and network-type analyses, in single nucleotide variant (SNV)- and copy number variation (CNV)-based analyses [20]. However, the role of the AI cannot be overestimated. It may be a tool, not a goal. Blind relying on the AI in wide-association studies may end in overlooking important association and artificial character of other dependencies.

Rich metadata, especially collected on an ongoing basis throughout the life of an individual, can make biobanks convenient tools of researching different types of epigenetic regulation and predisposition to a considerable number of infectious diseases.

## CONCLUSION

The Global Biobank Meta-analysis Initiative has large potential for assisting health specialists in research and treatment of hereditary and multi-factor diseases. However, a health care-related researcher or clinician should be well-prepared to the GMBI’s disadvantages discussed hereinabove. It should be always remembered that, for medicine, genetic findings, whichever fascinating and attractive they may seem, are merely an instrument, whatever useful it may be. This instrument can be added to the broad repository of global health research methods, but its importance should not be overestimated, nor absolutised.
